# *Salmonella* Infection Upregulates the Leaky Protein Claudin-2 in Intestinal Epithelial Cells

**DOI:** 10.1371/journal.pone.0058606

**Published:** 2013-03-11

**Authors:** Yong-guo Zhang, Shaoping Wu, Yinglin Xia, Jun Sun

**Affiliations:** 1 Department of Biochemistry, Rush University, Chicago, Illinois, United States of America; 2 Department of Biostatistics and Computational Biology, University of Rochester, Rochester, New York, United States of America; 3 Department of Medicine, Gastroenterology and Hepatology Division, University of Rochester, Rochester, New York, United States of America; Emory University School of Medicine, United States of America

## Abstract

**Background:**

Tight junctions seal the space between adjacent epithelial cells. Mounting evidence suggests that tight junction proteins play a key role in the pathogenesis of human disease. Claudin is a member of the tight junction protein family, which has 24 members in humans. To regulate cellular function, claudins interact structurally and functionally with membrane and scaffolding proteins via their cytoplasmic domain. In particular, claudin-2 is known to be a leaky protein that contributes to inflammatory bowel disease and colon cancer. However, the involvement of claudin-2 in bacterial infection in the intestine remains unknown.

**Methods/Principal Findings:**

We hypothesized that *Salmonella* elevates the leaky protein claudin-2 for its own benefit to facilitate bacterial invasion in the colon. Using a *Salmonella*-colitis mouse model and cultured colonic epithelial cells, we found that pathogenic *Salmonella* colonization significantly increases the levels of claudin-2 protein and mRNA in the intestine, but not that of claudin-3 or claudin-7 in the colon, in a time-dependent manner. Immunostaining studies showed that the claudin-2 expression along the crypt-villous axis postinfection. *In vitro*, *Salmonella* stimulated claudin-2 expression in the human intestinal epithelial cell lines SKCO15 and HT29C19A. Further analysis by siRNA knockdown revealed that claudin-2 is associated with the *Salmonella*-induced elevation of cell permeability. Epithelial cells with claudin-2 knockdown had significantly less internalized *Salmonella* than control cells with normal claudin-2 expression. Inhibitor assays demonstrated that this regulation is mediated through activation of the EGFR pathway and the downstream protein JNK.

**Conclusion/Significance:**

We have shown that *Salmonella* targets the tight junction protein claudin-2 to facilitate bacterial invasion. We speculate that this disruption of barrier function contributes to a new mechanism by which bacteria interact with their host cells and suggests the possibility of blocking claudin-2 as a potential therapeutic strategy to prevent bacterial invasion.

## Introduction

Intestinal tight junctions (TJs) seal the space between adjacent epithelial cells, which serve as a barrier, provide structure, and play a role in host defense. Many TJ proteins are known to tighten the cell structure and maintain a barrier [1]. In contrast, claudin-2 is a leaky protein that plays an opposing role to other TJ proteins. Increased claudin-2 in epithelial cells correlates with increased cell permeability. Moreover, recent evidence demonstrates that claudin-2 is involved in many signaling pathways, including vitamin D receptor, EGFR, and JNK signaling pathways, and it contributes to inflammatory bowel disease and colon cancer [2], [3], [4], [5], [6].

Enteric bacterial pathogens, such as *Salmonella*, can hijack their host cell machinery by altering the structure and function of the TJ barrier to facilitate bacterial invasion and infection [7]. These effects may result from direct modification of TJ proteins, such as occludin, claudin, and ZO-1, or by alteration of the perijunctional actomyosin ring during invasion and infection [7], [8]. However, it is unknown how the leaky protein claudin-2 is influenced by *Salmonella* infection.

We hypothesized that pathogenic *Salmonella* elevates the leaky protein claudin-2 for its own benefit to facilitate bacterial invasion in the colon. In this study, we used a *Salmonella*-colitis mouse model and cultured intestinal epithelial cells. We found that pathogenic *Salmonella* colonization increased claudin-2 mRNA and protein expression, but not that of claudin-3 or 7, *in vivo* and *in vitro*. Epithelial cells with claudin-2 knockdown had significantly less internalized *Salmonella* than control cells with normal claudin-2 expression. Inhibitor assays demonstrated that this regulation is mediated through activation of the EGFR pathway and the downstream protein JNK. The resulting data indicate that claudin-2 was upreguated by *Salmonella* during intestinal infection.

## Results

### *Salmonella* Infection Induces Elevated Claudin-2 in the Colon

**Figure 1:**
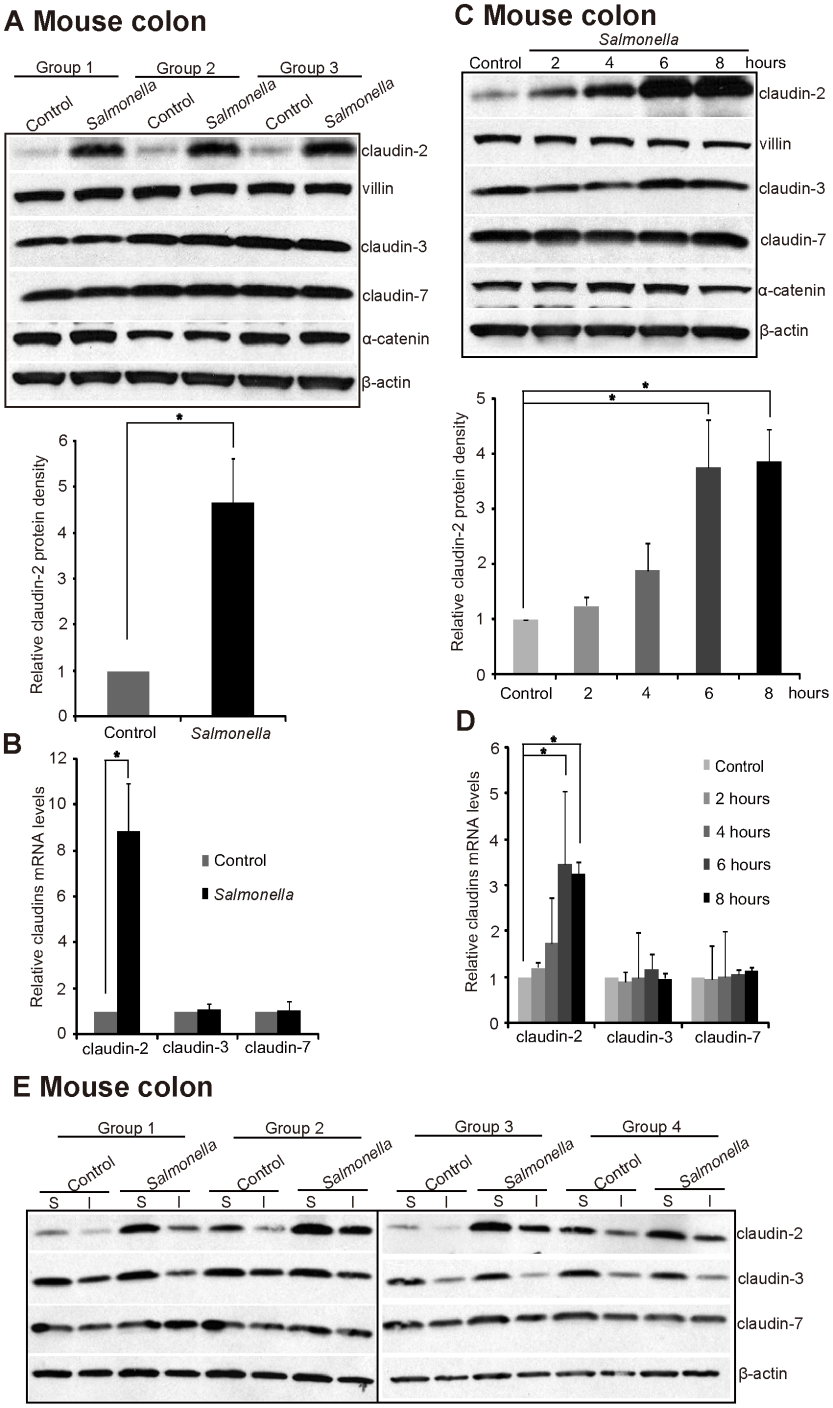
Pathogenic wild-type (WT) *Salmonella* increases claudin-2 mRNA and protein expression in colon epithelial cells *in vivo*. (A) Claudin-2 protein level and (B) Claudin-2 mRNA level in mice 8 hours post *Salmonella* infection. Mice were infected with *Salmonella* for 8 hours and intestinal epithelial cells were harvested for immunoblot and real-time PCR. Data are expressed as the mean ± SD. *P<0.05. n = 3. (C) and (D) *Salmonella*-induced claudin-2 protein and mRNA expression in a time-dependent manner. Mice were infected with *Salmonella* for the indicated duration and colonic epithelial cells were harvested for immunoblot and real-time PCR. Data are expressed as the mean ± SD. *P<0.05. n = 3 mice. (E). Pathogenic *Salmonella* increases claudin-2 protein expression in TritonX-100 soluble and insoluble fractions in colon epithelial cells. n = 4 mice. Mice were infected with *Salmonella* for 8 hours and intestinal epithelial cells were harvested for immunoblot.

We determined whether bacteria modulate claudin-2 expression using streptomycin-pretreated C57BL6 mice colonized with wild-type pathogenic *Salmonella* ATCC 14028s. Streptomycin treatment is known to diminish the intestinal flora and to render the mice susceptible to intestinal colonization by various microorganisms. In previous studies, we have used streptomycin-pretreated *Salmonella*-colitis mice to understand host-pathogen interactions [9], [10]. Here, we found that claudin-2 in the colon was significantly increased by pathogenic wild-type *Salmonella* (Fig. 1A). In contrast, the TJ proteins claudin-3 and 7 were not altered in the *Salmonella*-infected colon. Villin was used as an internal marker for the epithelial cells (Fig. 1A). We further found that the claudin-2 mRNA level in the colon was upregulated by *Salmonella* infection *in vivo*, whereas the other TJ proteins, claudin-3 and 7, were not changed by *Salmonella* infection (Fig. 1B).

To determine whether claudin-2 is changed in the early stage of *Salmonella* infection, we tested the expression of claudin-2 in the colon 2, 4, 6, and 8 hours postinfection. Using RT-PCR, we investigated mRNA expression of claudin-2, 3, and 7 in colon. The transcriptional levels of claudin-2 were significantly changed by wild type (WT) *Salmonella* 4 and 8 hours postinfection (Fig. 1C), whereas claudin-3 and 7 were not changed by WT *Salmonella*. Furthermore, we found that WT *Salmonella* significantly increased the total amount of claudin-2 protein in the colon after bacterial colonization for 6 hours (Fig. 1D). Protein lysates collected from mouse colon were performed using TritonX-100 buffer. The insoluble fraction of this lysates may contain a considerable amount of claudin-2. Therefore, we measured the claudin-2 protein levels from both control and *Salmonella* infected mice with Triton soluble and insoluble fractions (Fig.1E). In 4 replicated groups, we clearly showed that claudin-2 protein was increased in both soluble and insoluble fractions after *Salmonella* infection (Fig.1E). Taken together, our data showed that claudin-2 is elevated in the colon in the early stage of *Salmonella* infection.

### Distribution of Claudin-2 Protein in the *Salmonella*-infected Mouse Colon *in vivo*

**Figure 2:**
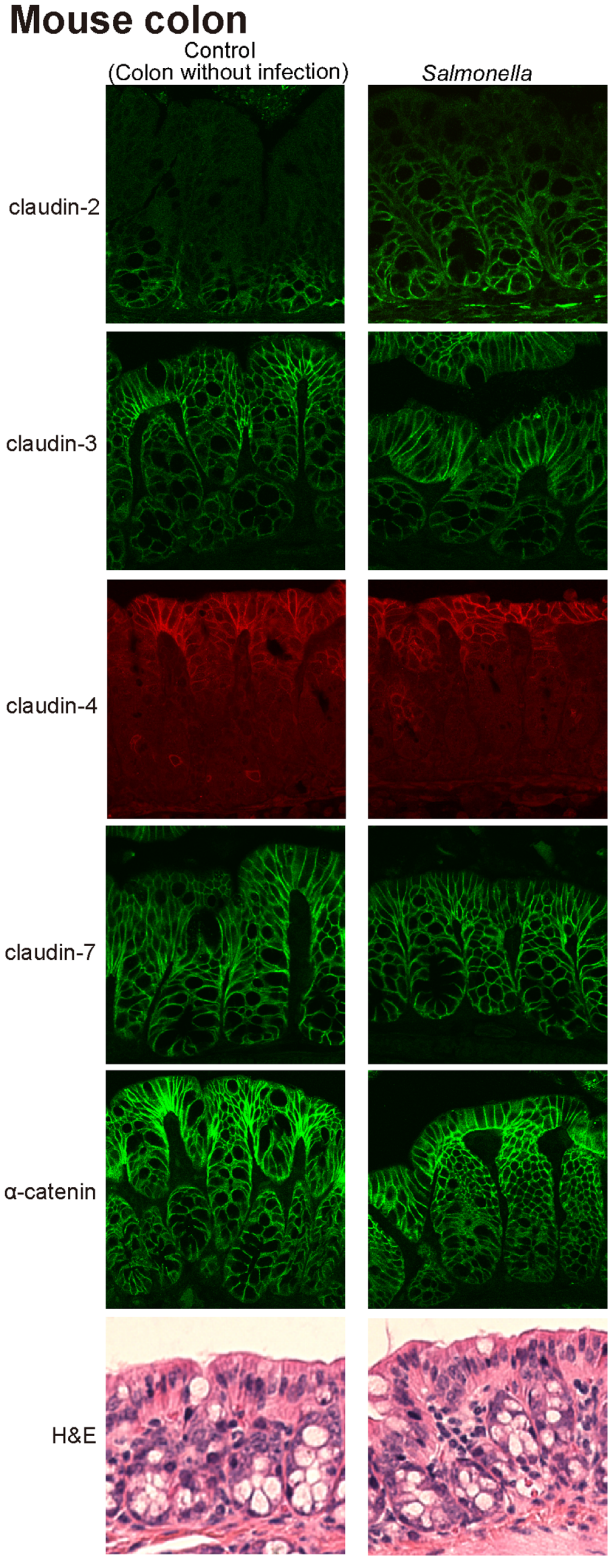
*Salmonella*-induced expression of claudin-2 along the crypt-villous axis *in vivo.* Immunostaining of mouse colon was performed 8 hours after mouse infection with *Salmonella*. Tissues were fixed, stained with claudin-2, 3, 4, 7, and α-catenin antibodies, and then stained with secondary antibodies. We found that *Salmonella* colonization in mice not only increased claudin-2 staining at the bottom of the crypts but also induced claudin-2 expression in the middle and top of the crypts. Images for each protein are shown from a single experiment and representative of three separate experiments. n = 3 mice/group.

Claudin-2 is known to be a TJ protein in epithelial cells. We investigated the distribution of claudin-2 in the *Salmonella*-infected mouse colon. We found that, in the normal colon, claudin-2 was located at the bottom of the epithelium. Pathogenic *Salmonella* colonization in mice not only increased claudin-2 staining at the bottom of the crypts, but also induced claudin-2 expression along the crypt-villous axis (Fig. 2, claudin-2). The distributions of claudin-3, 4, 7, and the cell adhesion protein α-catenin were not changed by WT *Salmonella* colonization in the colon. The H&E data showed intact epithelial structures 8 hours postinfection (the early stage of *Salmonella* infection). Taken together, our data indicate *Salmonella*-induced expression of claudin-2 along the crypt-villous axis in colon.

### *Salmonella* Infection Induces Elevated Claudin-2 in a Human Colonic Epithelial Cell Line

**Figure 3:**
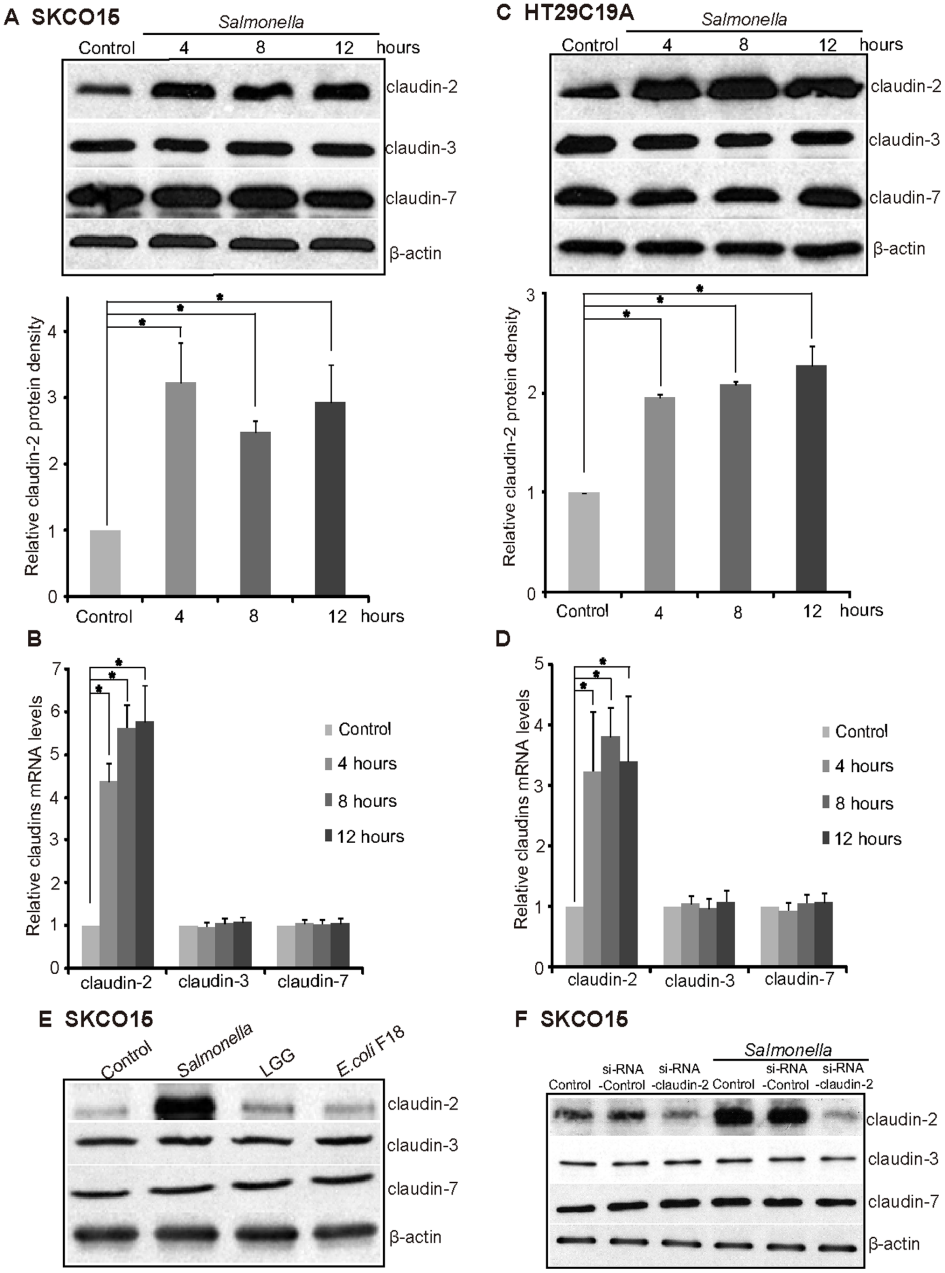
Pathogenic *Salmonella* increases claudin-2 mRNA and protein expression in human colonic epithelial cell line. (A) Claudin-2 protein level and (B) Claudin-2 mRNA level were upregulated in the human colonic epithelial cell line SKCO15. Cells were incubated with *Salmonella* WT for 30 minutes, washed, and incubated in fresh DMEM for the indicated duration. *P<0.05. n = 3 separate experiments. (C) Claudin-2 protein level and (D) Claudin-2 mRNA level were upregulated in the human colonic epithelial cell line HT29C19A. (E) Non-pathogenic bacteria, such as commensal bacterial *E. coli* F18 and probiotics L*GG*, did not alter claudin-2 protein expression in the human colonic epithelial SKCO15 cells. (F) Pathogenic *Salmonella*-induced claudin-2 protein was reduced by using siRNA against claudin-2. SKCO15 cells were pretreated with claudin-2 siRNA for 72 hours (40 nM) and then colonized with *Salmonella* for 30 minutes and incubated for 4 hours.

To look at the generality of our observation and the potential molecular mechanisms for claudin-2 in host-bacterial interactions, we investigated the response of the human colonic epithelial cell lines SKCO15 and HT29C19A. We had to use these cell lines because 1) SKCO15 is an intestinal cell model for TJ studies [11], [12] and 2) there is no non-cancer and non-transformed human colon cell line available in the TJ field. Claudin-2 was increased by pathogenic *Salmonella* after colonization for 4 to 12 hours, whereas claudin-3 and 7 were not changed in the SKCO15 (Fig. 3A) or the HT29C19A cells (Fig. 3C). At the mRNA level, claudin-2 was upregulated by *Salmonella* 4, 8, and 12 hours postinfection (Fig. 3B and Fig. 3D), whereas claudin-3 and 7 were not changed by *Salmonella*.

To test if the response of claudin-2 is through pathogenic *Salmonella*, we treated SKCO15 cells with human commensal bacterial *E. coli* F18 and probiotic strain *Lactobacillus rhamnosus GG* (Fig. 3E). However, we did not see the similar alternation of claudin-2. In a lost functional analysis, we knocked down the expression of claudin-2 by siRNA. As shown in Fig. 3F, in the cells with low claudin-2 expression, *Salmonella* was unable to increase the expression of claudin-2. In contrast, claudin-2 was increased by *Salmonella* infection in cells without siRNA or with control siRNA.

### *Salmonella* Colonization Altered the Distribution of Claudin-2 but not claudin-7 *in vitro*

**Figure 4:**
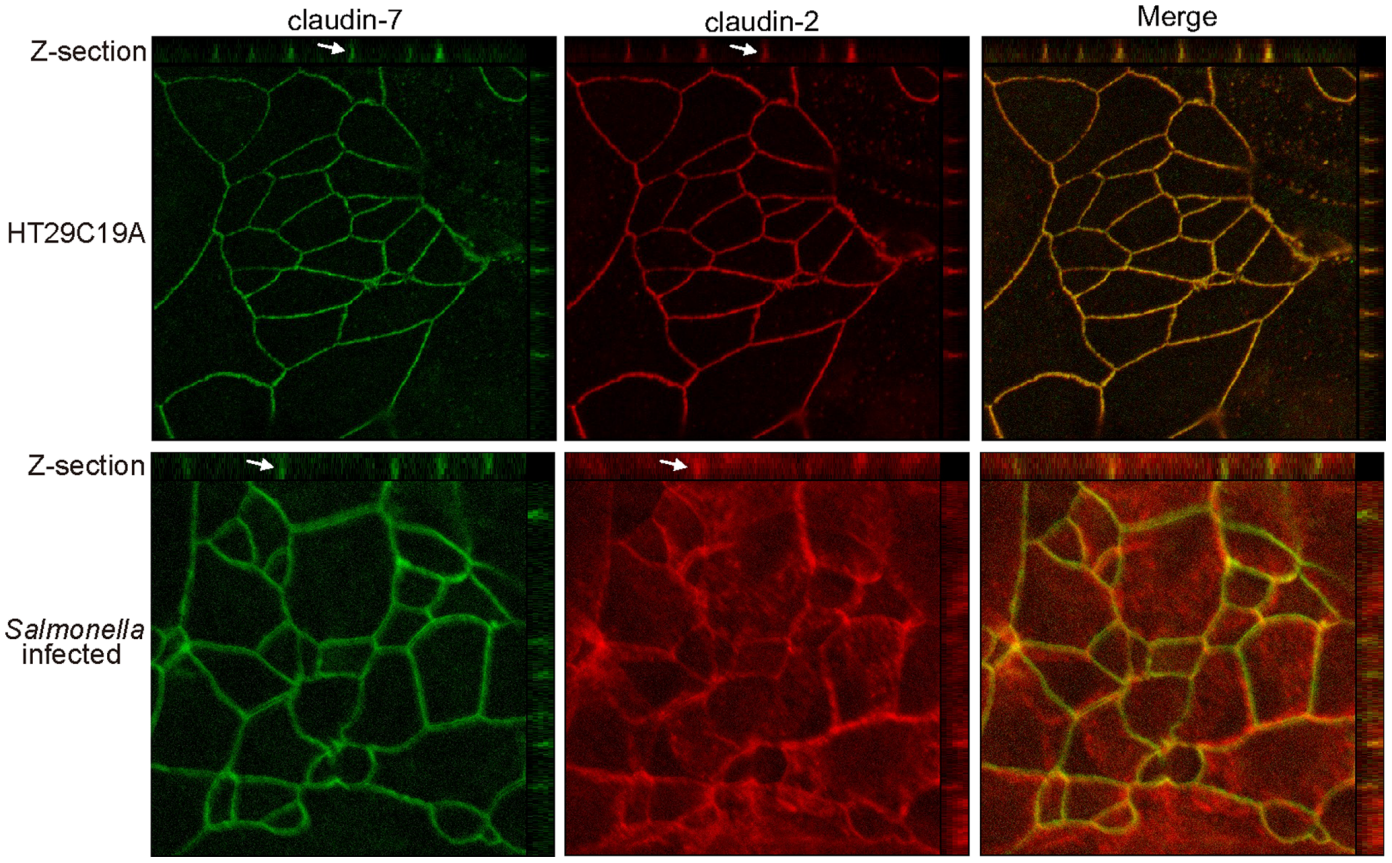
*Salmonella* colonization altered the distribution of claudin-2 but not that of claudin-7 *in vitro*. HT29C19A cells were colonized with *Salmonella* for 30 minutes followed by incubation for 4 hours. Immunostaining showed that the pathogenic *Salmonella* modulated the junctional localization of claudin-2.

We further examined whether *Salmonella* colonization altered the distribution of claudin-2 *in vitro*. Human epithelial cells, HT29C19A, colonized with *Salmonella* were analyzed for the location of claudin-2 and 7 (Fig. 4). In the control monolayer, claudin-2 and 7 were restricted to the cell borders in a smooth arc-like nature. In *Salmonella* treated cells, there was a striking disorganization of the transmembrane protein claudin-2, and the protein also expanded into the cytosolic region. Furthermore, the smooth arc-like claudin-2 profile was transformed into a complex series of irregular undulations (Fig. 4). Immunostaining of claudin-2 became thinner and more sinuous. The ring-like structure of claudin-2 was disrupted (Fig. 4, Z section). In contrast, we did not observe a similar change in claudin-7 in the *Salmonella-*treated cells. Overall, our immunofluorescent data suggest that *Salmonella* modulated the localization of claudin-2 within the junction.

### *Salmonella* Induces an Increase in Cell Permeability *in vitro* and *in vivo*

**Figure 5:**
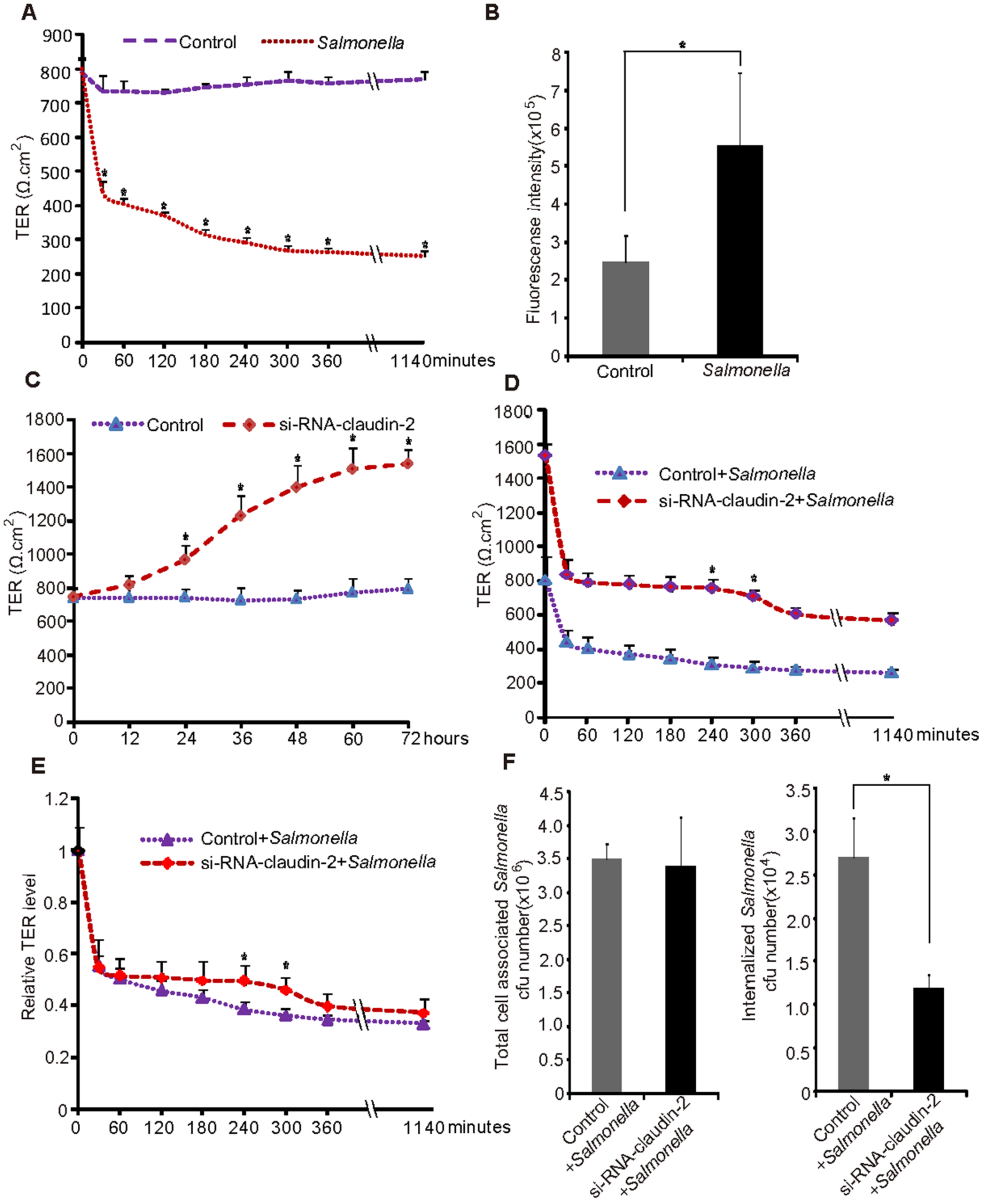
*Salmonella* increases cell permeability i*n vitro and in vivo* by inducing claudin-2 expression. (A) *Salmonella* increased permeability in human colonic epithelial cells. SKCO15 cells were colonized with *Salmonella* for 30 minutes and then incubated for the indicated times. (B) *Salmonella* increased mouse intestine permeability *in vivo*. Mice were infected with *Salmonella* for 24 hours, and then mouse blood samples were collected for fluorescence intensity measurement. Data are expressed as the mean ± SD. *P<0.05. n = 3 mice/group. (C) Claudin-2 knockdown in SKCO15 cells significantly enhanced TER of intestinal epithelial cells. SKCO15 cells were pretreated with claudin-2 siRNA (40 nM) for the indicated times, and then TER values were measured. (D) Claudin-2 is associated with the *Salmonella*-induced cell permeability increase. Pathogenic *Salmonella* cannot significantly decrease TER in those cells with lower claudin-2 expression compared to those cells with normal claudin-2 expression. SKCO15 cells were pretreated with claudin-2 siRNA for 72 hours (40 nM), colonized with *Salmonella* for 30 minutes, incubated for the indicated times, and then TER values were measured. (E) The ratio of relative TER change in cells in intestinal epithelial cells treated with or without claudin-2 siRNA. Values represent the ratio change in TER values compared to baseline TER value of each cell group at 0 minute (set as 1). Data are expressed as the mean ± SD. *P<0.05. n = 3. (F) Number of associated/invasive bacteria in intestinal epithelial cells treated with claudin-2 siRNA. Cells treated with claudin-2 siRNA had a significantly less number of invasive bacteria. Data are expressed as the mean ± SD. *P<0.05. n = 3 separate experiments.

Transepithelial resistance (TER) is a measure of intestinal epithelial integrity [13], [14]. We speculated that *Salmonella* induces elevated claudin-2 as a means to increase cell permeability and bacterial benefit in the intestine. We thus assessed the TER of the epithelial cells before or after the bacterial colonization. Cells were colonized with *Salmonella* for 30 minutes and washed. The TER of the monolayers was measured for 1140 minutes after switching to fresh media containing gentamicin to prevent further bacterial growth. TER decreased significantly for 30 minutes after *Salmonella* colonization and remained depressed for over 1140 minutes (Fig. 5A). *In vivo*, we also observed increased intestinal permeability 24 hours post *Salmonella* infection (Fig. 5B).

### Claudin-2 is Associated with the *Salmonella*-induced Increased Cell Permeability

To further investigate the molecular mechanisms, we blocked claudin-2 expression using siRNA. As shown in Fig. 5C, the knockdown of claudin-2 significantly enhanced the TER of intestinal epithelial cells. This observation is consistent with the role of claudin-2 as a leaky protein to reduce TER value and increase epithelial permeability. We then examined the effect of claudin-2 in *Salmonella*-infected cells by comparing the TER values between cells with normal and low claudin-2 expression levels. In our study, the TER change was observed for over 1140 minutes post *Salmonella* infection (Fig. 5D). Our data showed that the baseline TER (Ω.cm^2^) at 0 minute in cells without siRNA treatment was 810.3 Ω.cm^2^, whereas the TER values for the claudin-2 siRNA epithelial cells was 1535.7 Ω.cm^2^ at 0 minute incubation period. *Salmonella* induced reduction of the TER in both cells with normal claudin-2 expression (447.3 Ω.cm^2^) and with low cluadin-2 (840 Ω.cm^2^) at 30 minutes postinfection: *Salmonella* infection reduces TER values to around 50% initial values in both cells, regardless of the claudin-2 level. Although *Salmonella* induced low TER, the TER values at 240 and 300 minutes postinfection were significantly different between cells with normal claudin-2 expression and cells with low claudin-2 (Fig. 5D). Figure 5E showed the reduction ratios of relative TER level postinfection: *Salmonella*-induced reduction ratio of the TER was not significantly different from the cells with normal claudin-2 expression level at 30, 60, 120, and 180 minutes postinfection (Fig.5E). Rather, *Salmonella*-induced TER change was significantly different at 240 and 300 minutes time points (Fig. 5E). Overall, claudin-2 knockdown cells had higher TER compared to the cells with normal claudin-2 level before and post *Salmonella* infection.

### Claudin-2 Expression is Associated with Bacterial Invasion

We speculated that *Salmonella* increases intestinal permeability by targeting claudin-2 and increasing cell permeability, thereby increasing bacterial invasion. To investigate the physiological relevance of *Salmonella-*induced claudin-2 in the bacterial-host interactions, we detected bacterial invasion in intestinal epithelial cells. We counted the numbers of *Salmonella* invading the cells with normal or low levels of claudin-2 protein. We found that epithelial cells with claudin-2 knockdown had significantly less internalized *Salmonella* than control cells with normal claudin-2 expression (Fig. 5E). We also examined the number of cell-associated bacteria, including bacteria adhered to and/or internalized into the epithelial monolayers. However, claudin-2 expression did not change the number of associated *Salmonella* in the host cells (Fig. 5E).

### *Salmonella*-induced Claudin-2 is Dependent on the JNK Pathway

**Figure 6:**
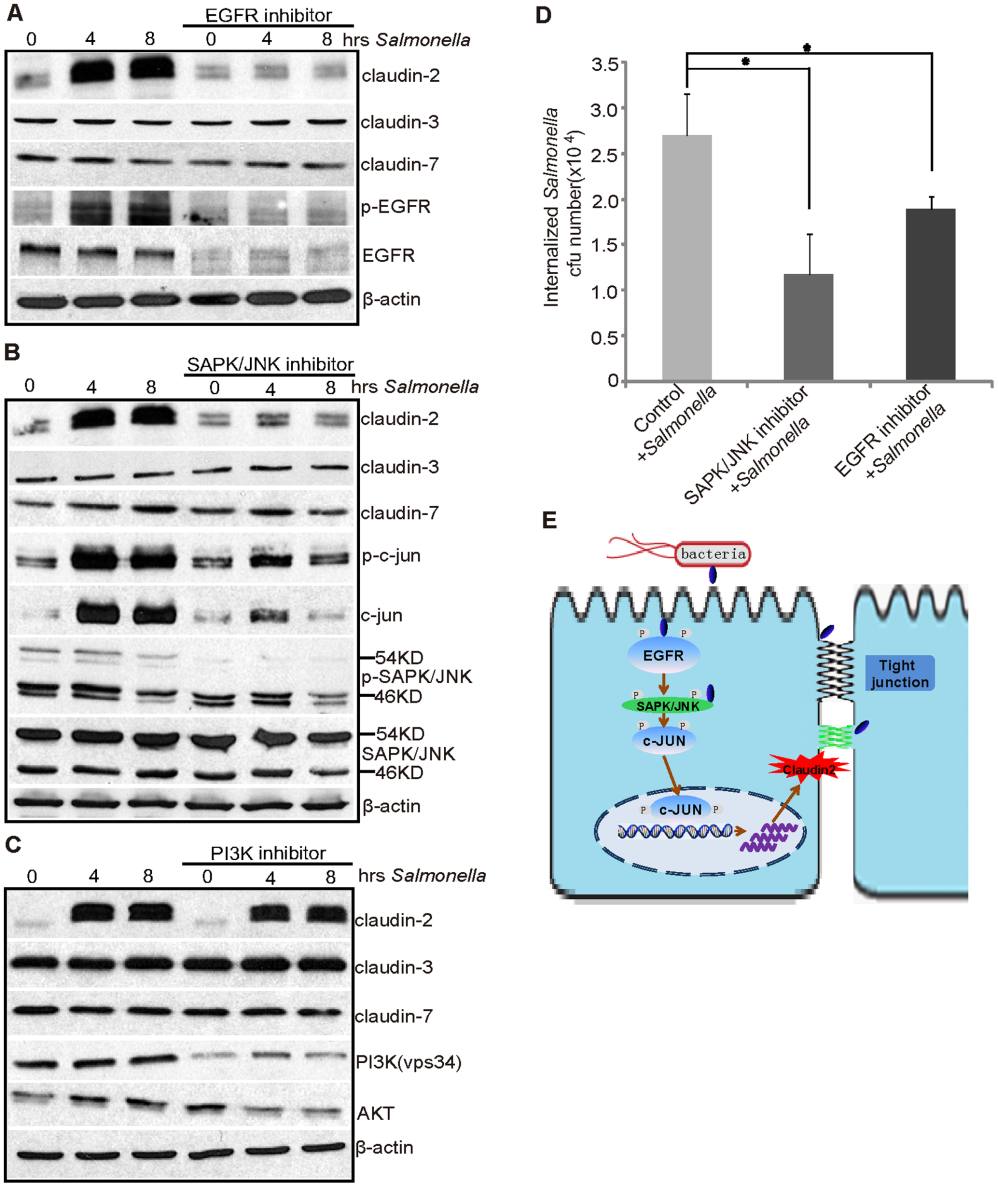
*Salmonella*-induced claudin-2 expression is dependent on the SAPK/JNK pathway. Claudin-2 protein expression induced by pathogenic *Salmonella* in SKCO15 cells was tested by different treatments in (A), (B) and (C). (A) Treatment with the EGFR inhibitor (Gefitinib, 50 ng/ml) for 1 hour, followed by colonization of *Salmonella* for 30 minutes and incubation for 4 hours. (B) Treatment with the SAPK/JNK inhibitor (SP600125, 50 µM) for 1 hour, followed by colonization of *Salmonella* for 30 minutes and incubation for 4 hours. (C) Treatment with the PI3K inhibitor (Wortmannin, 50 ng/ml) for 1 hour, followed by colonization of *Salmonella* for 30 min and incubation for 4 hours. (D) The number of invasive bacteria in the intestinal epithelial cells with the SAPK/JNK (SP600125) and the EGFR (Gefitinib) inhibitors. The SAPK/JNK and EGFR inhibitors can decrease the number of invasive bacteria. Data are expressed as the mean ± SD. *P<0.05. n = 3 separate experiments. (E) A diagram indicates that the *Salmonella*-induced claudin-2 expression is dependent on the EGFR and JNK pathways.

Claudin-2 expression is known to be regulated by EGFR signaling. Therefore, we hypothesized that inhibition of the EGFR pathway leads to claudin-2 protein disassembly. Using Gefitinib, an EGFR inhibitor, we blocked the change in *Salmonella*-induced claudin-2 expression, and there was no change in claudin-7 expression in cells with *Salmonella* or Gefitinib treatment (Fig. 6A). Interestingly, we also found that *Salmonella* treatment induced elevated phosphorylated-EGFR. Our data also shows that Gefitinib inhibits the expression of EFGR and phosphor-EGFR (Fig. 6A).

EGFR is an upstream regulator of the JNK pathway [15], . Inhibition of the JNK pathway is known to regulate the expressions of TJ proteins [17]. Therefore, we hypothesized that claudin-2 expression is regulated via the JNK pathway. In Fig. 6B, there was no elevated claudin-2 in cells first treated with the inhibitor SP600125 and then colonized by *Salmonella*. There was no change in claudin-7 expression in cells with *Salmonella* or with SP600125 treatment (Fig. 6B). C-Jun, a downstream target of the JNK pathway, was increased in cells with *Salmonella* infection, but blocked in the cells treated with the inhibitor postinfection. The total JNK was not altered in the cells with or without *Salmonella* treatment. Overall, our data indicate that *Salmonella*-induced claudin-2 was suppressed by blocking the EFGR and JNK pathways.

EGFR signaling also regulates the activity of PI3K [15]. However, we did not see the increased inhibition of the *Salmonella*-induced claudin-2 in the cells pretreated with the PI3K inhibitor Wortmannin (Fig. 6C). These data indicate that the effect of *Salmonella*-induced claudin-2 is dependent on EGFR and JNK, but not PI3K.

We further tested the association of *Salmonella* invasion and claudin-2 expression through the EGFR and its downstream JNK pathways. We did find that cells treated with the EGFR or JNK inhibitors had significantly less bacterial invasion compared to the cells without the inhibitor treatment. Thus, our data suggest that *Salmonella* targets claudin-2 and facilitates pathogenic enteric bacterial invasion (Fig. 6D).

## Discussion

In the current study, we demonstrate that *Salmonella* take advantage of the leaky TJ protein claudin-2 by increasing cell permeability and facilitating pathogenic enteric bacterial invasion. There is reduced *Salmonella* invasion in the claudin-2 knockdown cells compared to the cells with normal claudin-2. Blocking the JNK and EGFR signaling pathways is able to protect cells from bacterial invasion (Fig. 6E).

Our study reports that *Salmonella* invasion regulates leaky protein claudin-2 expression in the intestine. Previous studies showed that enteric bacterial pathogens can directly modify TJ proteins, such as occludin, claudins, and ZO-1, or by alter the perijunctional actomyosin ring during invasion and infection [7], [8]. These TJ proteins are known to tighten the epithelial structure and reduce cell permeability. However, it is unknown whether claudin-2 is influenced by *Salmonella* infection. Claudin-2 is known as a ‘leaky’ claudin that forms a paracellular water channel and mediates paracellular water transport in leaky epithelia [2], [18], [19], [20], [21]. Based on our current data (Fig. 5C), knockdown of claudin-2 alone was able to increase the TER of the colonic epithelial cells. It indicates the critical function of claudin-2 in maintaining epithelial permeability. *Salmonella* was able to reduce the TER at the early stage of bacterial-epithelial interaction, regardless of the level of claudin-2. We recognize the complex of the TJs and their regulation in bacterial infection. Our results suggest that claudin-2 is one of the TJ proteins contributing to alteration of the cell permeability during bacterial invasion in intestinal epithelial cells. Blocking claudin-2 expression could reduce bacterial invasion. However, knocking down claudin-2 was not sufficient enough to block the early influence of *Salmonella* on epithelial permeability. At 240 and 300 minutes after colonization, TER was significantly less reduced in the cells with low claudin-2 expression (Fig. 5D). Claudin-2 knockdown cells colonized with the *Salmonella* had relatively higher TER compared to the cells with normal claudin-2 level. These data indicate that knockdown of claudin-2 in epithelial cells may delay the *Salmonella*-induced increased cell permeability.

Previously, we have reported that a bacterial effector AvrA stabilizes cell permeability and expression of occludin-1, claudin-1, and ZO-1 in intestinal epithelial cells [8]. It is unclear whether claudin-2 is influenced by the AvrA expression. Future studies will explore this question. Taken together, both leaky and regular TJ proteins are involved in altering the structure and function of the intestinal barrier during bacterial invasion and infection.

Changes to claudin-2 are associated with many intestinal diseases, including active IBD [2], [4]. Peritonitis induced a threefold increase in intestinal permeability, which is associated with increased claudin-2 expression and a change in subcellular localization [22]. A recent study suggests that the HIV infected intestine had increased claudin-2 that resulted in leaky gut and microbial translocation [23]. It would be interesting to further study how claudin-2 contributes to chronic bacterial infection and inflammation in the intestine *in vivo*.

Preserving or restoring the barrier functions of the epithelial cells is a therapeutic strategy to prevent and treat infectious diseases. Probiotic treatment is shown to decrease claudin-2 expression and enhance epithelial cell barrier function [24]. Defective epithelial barrier function has been implicated in IBD and can predict relapse during clinical remission [2], [4], [25], [26], [27], [28], [29]. Our data also demonstrated that inhibitors blocking the JNK and EGFR signaling pathways are able to protect cells from *Salmonella* invasion through claudin-2. We speculate that this disruption of barrier functions contributes to a new mechanism by which the bacteria interacts with the host cells and suggests a possible use of blocking claudin-2 as a potential therapeutic strategy to prevent bacterial invasion and perhaps inflammation.

## Materials and Methods

### Ethics Statement

All animal work was approved by the University of Rochester University Committee on Animal Resources (UCAR) committee (UCAR 2007-065). Euthanasia method was sodium pentobarbital (100 mg per kg body weight) I.P. followed by cervical dislocation. Postinfection, the animals were observed closely and if any sign of discomfort such as unable to ambulate well enough to maintain hydration and caloric intake or greater than 10% weight loss is seen, the animal was euthanized immediately.

### Bacterial Strain and Growth Condition

Bacterial strain used in this study was *Salmonella typhimurium* wild-type strain ATCC14028s. Non-agitated microaerophilic bacterial cultures were prepared as previously described [30].

### Cell Culture

Human epithelial SKCO15 [11], [12], CaCo2BBE and HT29C19A cells [8], [31] were established cells lines derived from human colonic tumor cells. Cells were maintained in DMEM supplemented with 10% fetal bovine serum (FBS), streptomycin-penicillin and L-glutamine. Monolayers of SKCO15, CaCo2BBE, and HT29C19A cells were grown on permeable supports (0.33 or 4.67 cm^2^, 0.4 mm pore. Costar, Cambridge, MA, USA), as described in the previous publications [7], [10], [11].

### Bacterial Colonization in the Polarized Epithelial Cells *in vitro*

Polarized human epithelial cells were colonized with equal numbers of the indicated bacteria for 30 minutes, washed with HBSS, and incubated in DMEM containing gentamicin (500 mg/ml) for the times indicated in our previous studies [9], [30]. The first 30-minute incubation allowed bacteria to contact the surface of the epithelial cells and inject the effectors in the host cells. After extensive HBSS washing, the extracellular bacteria were washed away. Incubation with gentamicin inhibited the growth of bacteria.

### Streptomycin Pre-treated Mouse Model

Animal experiments were performed using specific-pathogen-free female C57BL/6 mice (Taconic, Hudson, NY, USA) that were 6–7 weeks old, as previously described [8], [10], [31]. The protocol was approved by the University of Rochester University Committee on Animal Resources (UCAR). Water and food were withdrawn 4 hours before oral gavage with 7.5 mg/mouse of streptomycin (100 µl of sterile solution or 100 µl of sterile water in control). Afterwards, animals were supplied with water and food ad libitum. Twenty hours after streptomycin treatment, water and food were withdrawn again for 4 hours before the mice were infected with 1×10^7^ CFU of *S. typhimurium* (100 µl suspension in HBSS) or treated with sterile HBSS (control). Eight hours after infection, mice were sacrificed, and tissue samples from the intestinal tracts were removed for analysis.

### Mouse Colonic Epithelial Cells

Mouse colonic epithelial cells were collected by scraping the mouse colon, including the proximal and distal regions. Cells were sonicated in lysis buffer (1% Triton X-100, 150 mM NaCl, 10 mM Tris pH 7.4, 1 mM EDTA, 1 mM EGTA pH 8.0, 0.2 mM sodium orthovanadate, protease inhibitor cocktail). The protein concentration was measured using BioRad Reagent (BioRad, Hercules, CA, USA). Protein lysates collected from mouse colon were performed using a TritonX-100 buffer. We also measured the protein levels of claudins from both control and *Salmonella* infected colon as Triton soluble and insoluble fractions.

### Immunoblotting

Mouse epithelial cells were scraped and lysed in lysis buffer (1% Triton X-100, 150 mM NaCl, 10 mM Tris pH 7.4, 1 mM EDTA, 1 mM EGTA pH 8.0, 0.2 mM sodium orthovanadate, protease inhibitor cocktail), and then the protein concentration was measured. Cultured cells were rinsed twice in ice-cold HBSS, lysed in protein loading buffer (50 mM Tris, pH 6.8, 100 mM dithiothreitol, 2% SDS, 0.1% bromophenol blue, 10% glycerol), and then sonicated. Equal amounts of protein were separated by SDS-polyacrylamide gel electrophoresis, transferred to nitrocellulose, and immunoblotted with primary antibodies. The following antibodies were used: anti-claudin-2, anti-claudin-3, anti-claudin-4, anti-claudin-7, anti-α-catenin, anti-VPS34 (Invitrogen, Grand Island, NY, USA), anti-p-SAPK/JNK, anti-SAPK/JNK, anti-p-c-jun, anti-p-c-jun, anti-p-EGFR, anti-EGFR, anti-AKT (Cell Signal, Beverly, MA, USA), anti-puma, anti-Villin (Santa Cruz Biotechnology Inc., Santa Cruz, CA, USA.), or anti-β-actin (Sigma-Aldrich, Milwaukee, WI, USA.) antibodies and were visualized by ECL (Thermo Scientific, Rockford, IL, USA). Membranes that were probed with more than one antibody were stripped before re-probing.

### Immunofluorescence Staining

Cultured epithelial cells HT29C19A were incubated with equal numbers of the indicated bacteria for 30 minutes and washed with HBSS. Immunofluorescent labeling of cells grown on inserts was performed as follows: cells were fixed for 10 minutes in 1% paraformaldehyde in HBSS and then washed in HBSS. Fixed samples were incubated in blocking solution (2% bovine serum albumin, 1% goat serum in HBSS) for 1 hour, followed by 4°C overnight incubation with primary antibodies. After a 60-minute incubation with secondary antibodies, the inserts were mounted with SlowFade (Invitrogen, Grand Island, NY, USA) followed by a coverslip, and the edges were sealed to prevent drying. Specimens were examined with a Zeiss LSM 710 Laser Scanning confocal microscope.

Colonic tissues from the proximal and distal portions of the colon were freshly isolated and embedded in paraffin wax after fixation with 10% neutral buffered formalin. After preparation of the slides as described above [10], slides were incubated in 3% H_2_O_2_ for 20 minutes at room temperature to block endogenous peroxidase activity, followed by incubation for 1 hour in blocking solution (2% bovine serum albumin, 1% goat serum in HBSS) to reduce nonspecific background. The samples were incubated overnight with primary antibodies at 4°C. Samples were then incubated with secondary antibodies for 1 hour at room temperature. Tissues were mounted with SlowFade. Specimens were examined with a Zeiss LSM 710 Laser Scanning confocal microscope.

### TER Measurement

Cells were grown as monolayers on collagen coated polycarbonate membrane Transwell supports (0.33 or 4.67 cm^2^, 0.4 mm pore. Costar, Cambridge, MA, USA). Cells were colonized with equal numbers of the indicated bacteria for 30 minutes, washed with HBSS, and incubated in DMEM containing gentamicin (500 ug/ml, Invitrogen Corporation) for the time indicated. Transepithelial resistance (TER) was measured with an epithelial voltohmmeter under open-circuit conditions (EVOM, World Percision Instruments, Sarasota, FL, USA). Each measurement was performed in triplicate.

### Fluorescence Permeability *in vivo*

Streptomycin pre-treated mice were infected with bacterial strains for 24 hours. Fluorescein Dextran (Molecular weight 3000 Da, diluted in HBSS) was gavaged (50 mg/g mouse). Four hours later, mouse blood samples were collected by cardiac puncture. Fluorescence intensity of the plasma was measured on a fluorescent plate reader [8], [32].

### Claudin-2 siRNA

SKCO15 cells were grown as monolayers on collagen coated polycarbonate membrane Transwell supports. The cells were transfected with esiRNA human CLDN2 (Sigma-Aldrich Co., Sigma, St. Louis, MO, USA) or scrambled siRNA control (Santa Cruz Biotechnology Inc., Santa Cruz, CA, USA) using Lipofectamine 2000 (Invitrogen, Grand Island, NY, USA) in accordance with the manufacturer’s instructions. Scramble control siRNA was referred as "control siRNA" in Figures. After transfection for 72 hours, cells were colonized by *Salmonella* for 30 minutes, washed, and incubated for 4 hours in DMEM with Gentamicin (500 µg/ml), and then the levels of claudin-2 and β-actin were assessed by western blot.

### Real Time Quantitative PCR

Total RNA was extracted from epithelial cell monolayers or mouse colonic epithelial cells using TRIzol reagent (Invitrogen, Grand Island, NY, USA). The RNA integrity was verified by gel electrophoresis. RNA reverse transcription was done using the iScript cDNA synthesis kit (Bio-Rad, Hercules, CA, USA) according to the manufacturer's directions. The RT-cDNA reaction products were subjected to quantitative real-time PCR using the MyiQ single-color real-time PCR detection system (Bio-Rad) and iQ SYBR green supermix (Bio-Rad) according to the manufacturer's directions. All expression levels were normalized to β-actin levels of the same sample. Percent expression was calculated as the ratio of the normalized value of each sample to that of the corresponding untreated control cells. All real-time PCR reactions were performed in triplicate. All PCR primers were designed using Lasergene software (Table 1) (DNAStar, Madison, WI, USA).

**Table 1:** Real-time PCR primers.

Gene name	Primers
Human-claudin-2F	ACCTGCTACCGCCACTCTGT
Human-claudin-2R	CTCCCTGGCCTGCATTATCTC
Human-claudin-3F	CTGCTCTGCTGCTCGTGTCC
Human-claudin-3R	TTAGACGTAGTCCTTGCGGTCGTAG
Human-claudin-7F	CATCGTGGCAGGTCTTGCC
Human-claudin-7R	GATGGCAGGGCCAAACTCATAC
Human-β-actin-F	AGAGCAAGAGAGGCATCCTC
Human-β-actin-R	CTCAAACATGATCTGGGTCA
Mouse-claudin-2F	GCAAACAGGCTCCGAAGATACT
Mouse-claudin-2R	GAGATGATGCCCAAGTACAGAG
Mouse-claudin-3F	CCTGTGGATGAACTGCGTG
Mouse-claudin-3R	GTAGTCCTTGCGGTCGTAG
Mouse-claudin-7F	GCGACAACATCATCACAGCC
Mouse-claudin-7R	CCTTGGAGGAATTGGACTTGG
Mouse-β-actin-F	TGTTACCAACTGGGACGACA
Mouse-β-actin-R	CTGGGTCATCTTTTCACGGT

### *S. typhimurium* Invasion of Human Epithelial Monolayers

Infection of SKCO15 cells was performed by a previously described method [31]. Bacterial solution was added, and bacterial invasion was assessed after 30 minutes. Cell-associated bacteria, representing bacteria adhered to and/or internalized into the monolayers, were released by incubation with 100 ml 1% Triton X-100 (Sigma). Internalized bacteria were those obtained from lysis of the epithelial cells with 1% Triton X-100 30 minutes after the addition of gentamicin(500 µg/ml). For both cellassociated and internalized bacteria, 0.9 ml LB broth was added, and each sample was vigorously mixed and quantitated by plating for CFU on MacConkey agar medium.

### Statistical Analysis

Data are expressed as mean ± SD. Differences between two samples were analyzed by Student's t test. P-values of 0.05 or less were considered statistically significant. Differences among three or more groups were analyzed using ANOVA (SAS 9.2 version, SAS Institute Inc., Cary, NC, USA).
